# Growth and survival of heat-, cold-, and acid-stressed *Salmonella* Typhimurium and native bacteria on chicken wings during refrigerated storage and temperature abuse

**DOI:** 10.1016/j.psj.2026.107618

**Published:** 2026-08-15

**Authors:** Zhen Jia, Brenda Jovel Gonzalez, Stephen N. White, Lihan Huang, Ranju Kafle, Kathryn Owen, Shabarinath Srikumar, Amit Morey, Laura Garner, Landon Rohling, Arabella Jones, Cameron Smith

**Affiliations:** a Department of Poultry Science, Auburn University, Auburn, AL, 36849, USA; b U.S. Department of Agriculture, Agricultural Research Service, U.S. National Poultry Research Center, Athens, GA, 30605, USA; c U.S. Department of Agriculture, Agricultural Research Service, Eastern Regional Research Center, Wyndmoor, PA, 19038, USA; d Department of Biological Sciences, Auburn University, Auburn, AL, 36849, USA

**Keywords:** *Salmonella*, Cold stress, Heat stress, Acid stress, Chicken wings

## Abstract

During food processing, *Salmonella* is frequently exposed to environmental stresses that may alter its growth and survival, thereby influencing food safety and accuracy of microbial risk assessment. This study investigated the behavior of *Salmonella* Typhimurium (ST), unstressed (USST), heat-stressed (HSST), cold-stressed (CSST), and acid-stressed (ASST) on chicken wings and developed models to predict shelf life during storage at 4, 10, and 20°C. At all temperatures, native bacteria (NB) grew and were modeled using a no-lag phase (primary) model and Ratkowsky square-root (secondary) model for chicken wing shelf-life prediction. At 4 and 10°C, the ST populations, stressed or unstressed, gradually declined, whereas full growth curves were observed at 20°C. At 4°C, USST and stressed ST followed linear survival kinetics and the D value of CSST was 3-4 d longer than those of USST, HSST, and ASST. At 10°C, USST maintained a linear decline trend, whereas all stressed ST declined nonlinearly with shape parameters greater than 1. These results suggested that stressed ST may have potentially developed a defense mechanism during low-temperature storage. At 20°C, stress influenced ST’s lag phase, growth rate, and maximum population, particularly for ASST. These findings demonstrated that stress effects on ST behavior were temperature- and stress-dependent. The estimated ST kinetic parameters provide valuable information for understanding stress-induced changes in ST behavior and support future development of predictive models incorporating temperature and stress factors for microbial risk assessment.

## Introduction

Foodborne illnesses remain a major global public health issue. The World Health Organization (WHO) estimates that nearly 1 in 9 people in the world, approximately 866 million people, fall ill each year due to consumption of contaminated food (World Health Organization, 2026). Diarrheal diseases are among the most common foodborne illnesses, affecting 550 million people each year (World Health Organization, 2018). *Salmonella* is one of the four key pathogens causing diarrheal diseases, along with *Escherichia coli, Shigella* spp*., and Campylobacter* spp*.* (World Health Organization, 2018, World Health Organization, 2024). In the United States, *Salmonella* is the second leading cause of foodborne illnesses and the leading cause of hospitalizations and deaths from foodborne illness (CDC, 2024a; U.S. Food and Drug Administration, 2023). Every year, it is responsible for around 1.35 million infections, 26,500 hospitalizations, and 420 deaths (CDC, 2024a; U.S. Food and Drug Administration, 2023). *Salmonella* infections are commonly linked to eating contaminated food, with poultry products as a major source (U.S. Food and Drug Administration, 2023; World Health Organization, 2018), accounting for roughly 25% of *Salmonella* illnesses (CDC, 2024b). The United States Department of Agriculture - Food Safety and Inspection Service (USDA-FSIS) reported that about 125,000 *Salmonella* illnesses are associated with chicken products every year (Singer, 2025; USDA-FSIS, 2024a). Foodborne illnesses have resulted in a large amount of costs. According to the USDA’s Economic Research Service, the cost of foodborne illnesses from 31 major foodborne pathogens in 2023 was about $44.7 billion (Ahn, 2025). The cost of *Salmonella* ranked first, contributing approximately $17.1 billion (Ahn, 2025). Because *Salmonella* infections impose significant health and economic burdens, and poultry products are a major source, predicting pathogen risk in poultry products is critical for improving food safety and public health.

Temperature abuse often occurs in the food supply chain and is a primary factor promoting *Salmonella* growth and causing *Salmonella* infections. Conventional culture-based methods or rapid microbial techniques are generally the main tools for quantifying microbial growth in food. Conventional culture-based methods need on-site sampling and laboratory testing; they are labor-intensive and time-consuming, usually taking over 5-7 days (U.S. Department of Agriculture-Food Safety and Inspection Service, 2024b). Rapid testing methods provide faster results, but they also require specialized instruments and trained personnel. They cannot provide real-time estimates of bacterial contamination. Predictive modeling in microbiology, on the other hand, has emerged as a tool for risk prediction and assessment (Perez-Rodriguez and Valero, 2013). Numerous predictive models have been developed to describe the growth and survival of *Salmonella* in foods. Many models have been specifically developed for predicting the growth of *Salmonella* on raw chicken meat using different models, such as the Huang model (Milkievicz et al., 2020), Baranyi model (Noviyanti et al., 2024; Oscar, 2009a; Zhou et al., 2014), modified Gompertz model (Juneja et al., 2007; Oscar, 2007; Peruzzolo et al., 2024), neural network model (Oscar, 2009b, 2011, 2015), competitive Huang-Jameson effect model (Jia et al., 2020), and the modified Weibull model (Pradhan et al., 2012), using *Salmonella* strains not previously exposed to stressed conditions. However, *Salmonella* frequently experiences various environmental stresses in poultry processing, such as heat stress during soft and hard scalding, acid stress from sanitization operations, and cold stress during chilling (Marmion et al., 2022; Owens et al., 2010).

Exposure to stress may trigger *Salmonella* adaptations and cell defense mechanisms, leading to changes in its survival or growth behaviors (Alnahhas and Dunlop, 2023; Osaili et al., 2017 & 2021), enhancing its resistance to other stresses (Kim et al., 2021; Krüger et al., 2025; Lin et al., 2012; Wu et al., 2021; Xavier and Ingham, 1997), and potentially promoting its virulence (Nielsen et al., 2013). The effects of stressors on *Salmonella* have been previously reported in some studies. For example, Tiwari et al., 2004 reported that acid stress could enhance the survival of *Salmonella* compared to unstressed cells; Smet et al., 2015 found that the osmotic-stressed *Salmonella* exhibited a higher specific growth rate compared to unstressed cells; and Shah et al., 2013 reported that cold stress could significantly increase *Salmonella* survival. These findings suggest that stress may alter bacterial growth kinetics and survival potential. Consequently, existing models that were developed using unstressed *Salmonella* may be inadequate for describing the behavior of stressed *Salmonella*.

This study aimed to investigate the effects of cold, heat, and acid stresses on the growth and survival of *Salmonella* on chicken wings during storage at refrigerated and abuse temperatures. In addition, predictive models were developed to describe native bacteria (**NB**) growth and to predict chicken wing shelf life. This work provides insight into how environmental stresses affect *Salmonella* behavior in poultry products, which has important implications for poultry meat safety management, intervention strategies, and risk assessment.

## Methods and materials

### Materials and chemicals

Nalidixic acid sodium salt (**NA**) was purchased from Sigma-Aldrich Co. (Saint Louis, MO, USA). Brain heart infusion (**BHI**), tryptic soy agar (**TSA**), xylose lysine deoxycholate (**XLD**) agar, and buffered peptone water (**BPW**) were purchased from Hardy Diagnostics (Santa Maria, CA, USA). Rappaport-Vassiliadis (**RV**) broth was purchased from Neogen Corporation (Lansing, MI, USA). Glacial acetic acid was obtained from Mallinckrodt Chemicals (St. Louis, MO, USA). Since stress responses and growth kinetics may vary considerably among strains, a single strain of *Salmonella enterica* serovar Typhimurium ST4/74 (**ST**), which is one of the common *Salmonella* serotypes of concern associated with chicken products (U.S. Department of Agriculture, 2024), was studied in this work. ST stock culture was stored in BHI broth containing 10% glycerol at −80°C. Chicken wings were obtained from a local market. The samples were tested for the presence of indigenous *Salmonella* by using ten randomly selected wings. Each wing was placed in 100 mL RV broth for enrichment (U.S. Department of Agriculture-Food Safety and Inspection Service, 2024b) at 37°C for 24 h (Nair et al., 2023). The enrichment was streaked onto XLD plates supplemented with 0 - 200 ppm NA and incubated at 37°C for 24 h (Nair et al., 2023). Colonies displaying red with black centers on XLD plates and on XLD plates containing up to 100 ppm NA (data not shown) indicated the presence of indigenous *Salmonella* capable of surviving up to 100 ppm NA.

### NA-resistant salmonella strain preparation

To differentiate ST from native *Salmonella*, ST was induced to resist 200 ppm NA, which exceeds the tolerance of indigenous *Salmonella*. The induction was performed by sequentially subculturing in BHI broth containing increasing concentrations of NA (0, 10, 20, 30, 50, 80, 100, 150, and 200 ppm) (Fang et al., 2013). Cultures were incubated in a shaking incubator at 37°C for 24 h at 150 rpm. Once the NA resistance was induced and stabilized in broth, the stability of the NA-resistant ST strain was further confirmed on agar plates by plating on XLD plates containing 200 ppm NA (**XLD/NA**). NA-resistant ST culture demonstrating robust growth in both BHI broth and XLD/NA plate was stored in BHI broth supplemented with 200 ppm NA and 10% glycerol at −80°C as a stock culture for downstream experiments.

### Unstressed and stressed salmonella inoculum preparation

Prior to experiments, NA-resistant ST was revived by transferring a loopful of the stock culture into 10 mL BHI broth containing 200 ppm NA, followed by incubating at 37°C for 24 h with shaking at 150 rpm. The culture was streaked onto XLD/NA plates and incubated at 37°C for 48 h. A single colony of NA-resistant ST was then sub-cultured twice in 10 mL BHI broth containing 200 ppm NA and streaked on an XLD/NA plate under the same conditions. The NA-resistant *Salmonella* culture was regularly propagated and maintained on the XLD/NA plate and stored at 4°C.

***Unstressed ST (USST).*** A single colony of NA-resistant ST culture was inoculated into 10 mL fresh BHI broth containing 200 ppm NA and incubated at 37°C for 24 h at 150 rpm. Bacterial cells were harvested by centrifuging at 4,500 rpm for 15 min at room temperature, washed once with 10 mL sterilized 0.1% BPW, and finally resuspended in 5 mL 0.1% BPW. The suspension was serially diluted to approximately 5 log CFU/mL to serve as the USST inoculum.

***Cold-stressed ST (CSST).*** Unstressed NA-resistant ST cell pellet was resuspended in pre-chilled 5 mL BHI broth containing 200 ppm NA (4°C) and stored at 4°C for 2 h to induce CSST. This treatment was used to simulate the chilling step in commercial broiler processing (Emami et al., 2021). After storage, cells were then harvested using the same procedure for USST to prepare a CSST working culture of approximately 5 log CFU/mL.

***Heat-stressed ST (HSST).*** Unstressed NA-resistant ST cell pellet was resuspended in 5 mL BHI broth containing 200 ppm NA at room temperature and held at 57°C for 2.5 min in a water bath. This treatment was selected to represent heat stress associated with the soft scalding during broiler processing (Shung et al., 2022) while maintaining consistent and reproducible exposure conditions. Treated cells were harvested using the same procedure for USST to prepare an HSST working culture of approximately 5 log CFU/mL.

***Acid-stressed ST (ASST).*** Cells were prepared as described for USST. After washing, the NA-resistant ST cell pellet was resuspended in 5 mL BHI broth containing 200 ppm NA adjusted to pH = 5.00 ± 0.2 with glacial acetic acid and incubated at 37°C for 2 h (Wu et al., 2021). This treatment was used as a simplified laboratory model of processing-related acid stress. After treatment, cells were harvested using the same procedure for USST to prepare an ASST working culture of approximately 5 log CFU/mL.

The above stress conditions used to induce stressed ST were designed as controlled laboratory models to evaluate the effects of specific stress conditions on ST survival and subsequent behavior under standardized and reproducible conditions. These treatments were not intended to exactly replicate commercial poultry processing environments, where microorganisms may encounter dynamic temperature changes, variable exposure times, organic matter, and sequential or combined stresses.

### Sample inoculation, sampling, and microbiological analysis

Chicken wings (50 wings per temperature for 4 and 10°C, and 70 wings for 20°C) were aseptically placed on sterilized aluminum foil sheets in a biosafety cabinet and spot-inoculated under 5 different experimental scenarios: 1 negative control, 1 positive control, and 3 stressed ST groups. 10 wings were set for each scenario for 4 and 10°C, whereas 14 wings per scenario were used for 20°C. Negative control samples were inoculated with 1.0 mL 0.1% BPW. Positive control samples were inoculated with 1.0 mL USST inoculum. Stressed samples were inoculated with 1.0 mL of CSST, HSST, and ASST inoculum, respectively. Sterile L-shaped plastic spreaders were used to evenly distribute the inoculum on the wings. After inoculation, the wings were kept in the biosafety cabinet at room temperature for 30 min to allow *Salmonella* attachment (Nair et al., 2023). Subsequently, each wing was aseptically placed in a sterile VWR® sterile sample bag (17.8 cm width × 30.5 cm length; VWR International, Radnor, PA, USA) and stored in chambers at temperatures of 4, 10, and 20°C that products may be exposed to from farm to table. During the storage, one chicken wing sample in the sterile sampling bag per scenario per temperature was withdrawn from storage at each sampling time point (0, 2, 4, 6, 18, 22, 24, 27, 30, 42, 48, 54, 66, and 72 h for 20°C, and 0, 1, 3, 5, 7, 10, 15, 20, 25, and 30 d for 4 and 10°C). The samples were analyzed for microbiological analysis to enumerate bacterial populations by following the published microbiological analysis procedures in raw poultry products (Cano et al., 2022; Kataria et al., 2020; Thanissery and Smith, 2014; Brichta‐Harhay et al., 2008; U.S. Department of Agriculture-Food Safety and Inspection Service, 2021). Each sample was added with 100 mL sterilized BPW, and rinsing was performed by shaking manually for 2 min to detach bacteria. The rinsate, which was the BPW liquid collected after each chicken wing was rinsed/shaken in a sterile sample bag, from each sample was collected to determine the bacterial counts. The colony-forming-unit (**CFU**) of bacteria recovered from each rinsate was recorded as CFU per mL of rinsate and was used to represent bacterial growth and survival on chicken wings. For positive control and stressed samples inoculated with *Salmonella*, the rinsate of each sample were serially diluted using 0.1% BPW and plated on XLD/NA agar plates to quantify *Salmonella*. For negative control samples with no *Salmonella*, TSA agar plates were used to enumerate the population of viable native bacteria (**NB**). All plates were incubated at 37°C for 48 h in an aerobic chamber. The number of colonies was counted and converted to log CFU/ mL rinsate. The unit of log CFU/ mL rinsate for chicken parts (including chicken wings) is used by the USDA (USDA-FSIS, 2024c) and studies on chicken wings in the poultry field (Bourassa et al., 2021; Incili et al., 2020; Kataria et al., 2020; Thanissery and Smith, 2014). Three independent experimental replicates were performed for each temperature and inoculation scenario.

### Mathematical models and data analysis

***Growth of NB during storage.*** During storage between 4 and 20°C, NB grew readily in chicken wings, exhibiting only exponential and stationary phases, matching the characteristics of the no-lag phase model (Eq. (1)) (Fang et al., 2013; Jia et al., 2025). The USDA Integrated Pathogen Modeling Program (**IPMP**) (Huang, 2014) was used to estimate growth kinetic parameters. In Eq. (1), Y_0_, Y(t), and Y*_max_* represent the bacterial populations in ln CFU/mL rinsate at the initial time, time t, and the maximum population level, respectively; $\mu_{max}$ denotes the specific growth rate (ln CFU/mL rinsate per d or per h); and t is the storage time (d or h).

(1) $$Y\left(t\right)=Y_{0}+Y_{max}-ln\left[e^{Y_{0}}+\left(e^{Y_{max}}-e^{Y_{0}}\right)e^{-\mu_{max}t}\right]$$

The effect of temperature on the specific growth rate of NB was analyzed using the secondary Ratkowsky square-root model (Eq. (2), Ratkowsky et al., 1983). In the model, **K**, which is $\mu_{max}/2.303$, is the maximum growth rate (log CFU/mL rinsate per d or per h). The coefficients in Eq. (2) were obtained by linear regression in Excel. The T_0_ value was calculated from the intercept by dividing it with -a. To convert K back to $\mu_{max}$, it is necessary to multiply K with 2.303 for use in Eq. (1).

(2) $$\sqrt{K\;}=a\left(T-T_{0}\right)$$

***USST and stressed ST survival at 4 and 10***°***C.*** USST and stressed ST did not grow, but exhibited survival behaviors with declining populations on chicken wings during storage at 4 and 10°C. The observed data indicated that the populations of both USST and stressed ST decreased linearly at 4°C. At 10°C, USST continued to decline linearly, while stressed ST displayed nonlinear survival patterns. To elucidate and characterize the survival behaviors of USST and stressed ST, ST data at 4°C were fitted to a linear model (Eq. (3)), while ST data at 10°C were fitted to a Weibull model (Eq. (4); Mafart et al., 2002). Model parameters were estimated using the USDA-IPMP (Huang, 2014).

(3) $$y\left(t\right)=y_{0}-\frac{t}{D}$$

(4) $$y\left(t\right)=y_{0}-{\left(\frac{t}{D}\right)}^{\alpha }$$

In Eqs. (3) and (4), y(t), which is different from Y(t) in Eq. (1), represents the bacterial population in log CFU/mL rinsate at time t; y_0_ represents the initial bacterial population in log CFU/mL rinsate; D is the first decimal reduction time, d; and α is the shape parameter (α>1 means a concave-downward survival curve; α=1 indicates a linear survival curve; α<1 means a concave-upward survival curve) (González et al., 2009; Huang and Juneja, 2003).

***USST and stressed ST growth at 20***°***C.*** In contrast to 4 and 10°C, unstressed and stressed ST grew at 20°C. These cells exhibited three distinct phases: lag phase, exponential phase, and stationary phase, in contrast to NB. Accordingly, ST data at 20°C were fitted to a full growth model, the Huang model (Eq. (5); Huang, 2013). The USDA-IPMP (Huang, 2014) was used to determine the growth kinetic parameters: lag phase, specific growth rate, and maximum population.

(5) $$\left\{ \begin{array}{c} Y\left(t\right)=\;Y_{0}+Y_{max}-ln\left\{e^{Y_{0}}+\;\left[e^{Y_{max}-Y_{0}}\right]e^{-\mu_{max}B\left(t\right)}\right\}\\ B\left(t\right)=t+\;\frac{1}{\alpha }\ln\frac{1+\;e^{-\alpha \left(t-\lambda \right)}\;}{1+\;e^{\alpha \lambda }} \end{array}\right.$$

In Eq. (5), Y(t) represents the bacterial population in ln CFU/mL rinsate at time t; Y_0_ and Y*_max_* represent the initial and maximum bacterial population in ln CFU/mL rinsate, respectively; λ represents the lag phase (h); μ_max_ represents the specific growth rate (ln CFU/mL rinsate per h); and t is the storage time (h).

***Model validation.*** In this study, the linear, Weibull, and Huang models were employed to describe survival/growth kinetics, estimate parameters, and quantitatively characterize the behaviors of USST and stressed ST rather than for predictive purposes; therefore, validation was not performed for these models. The predictive performance of the developed no-lag phase model was validated using independent experimental data that were not used in model development. Model predictions were compared with the corresponding experimental data collected at each temperature. Model performance was assessed by the root-mean-square error (**RMSE**), which represents the difference between the predictive and experimental values (Eq. (6)). In Eq. (6), *y_obs_* and y*_pred_* are the observed and predicted bacterial counts (log CFU/mL rinsate) at each time point, *n* is the number of observed data points. In addition, residual errors, defined as the differences between predicted and observed values at each sampling time point, were analyzed to further evaluate model accuracy. The distribution of residual errors was analyzed using Python 3.12, with libraries NumPy, Pandas, seaborn, Matplotlib, and SciPy.

(6) $$RMSE=\;\sqrt{\frac{\sum_{i}^{n}{\left(y_{obs,i}-y_{pre,\;i}\right)}^{2}}{n}}$$

## Results and discussion

### Native bacteria (NB) growth

**Fig. 1:**
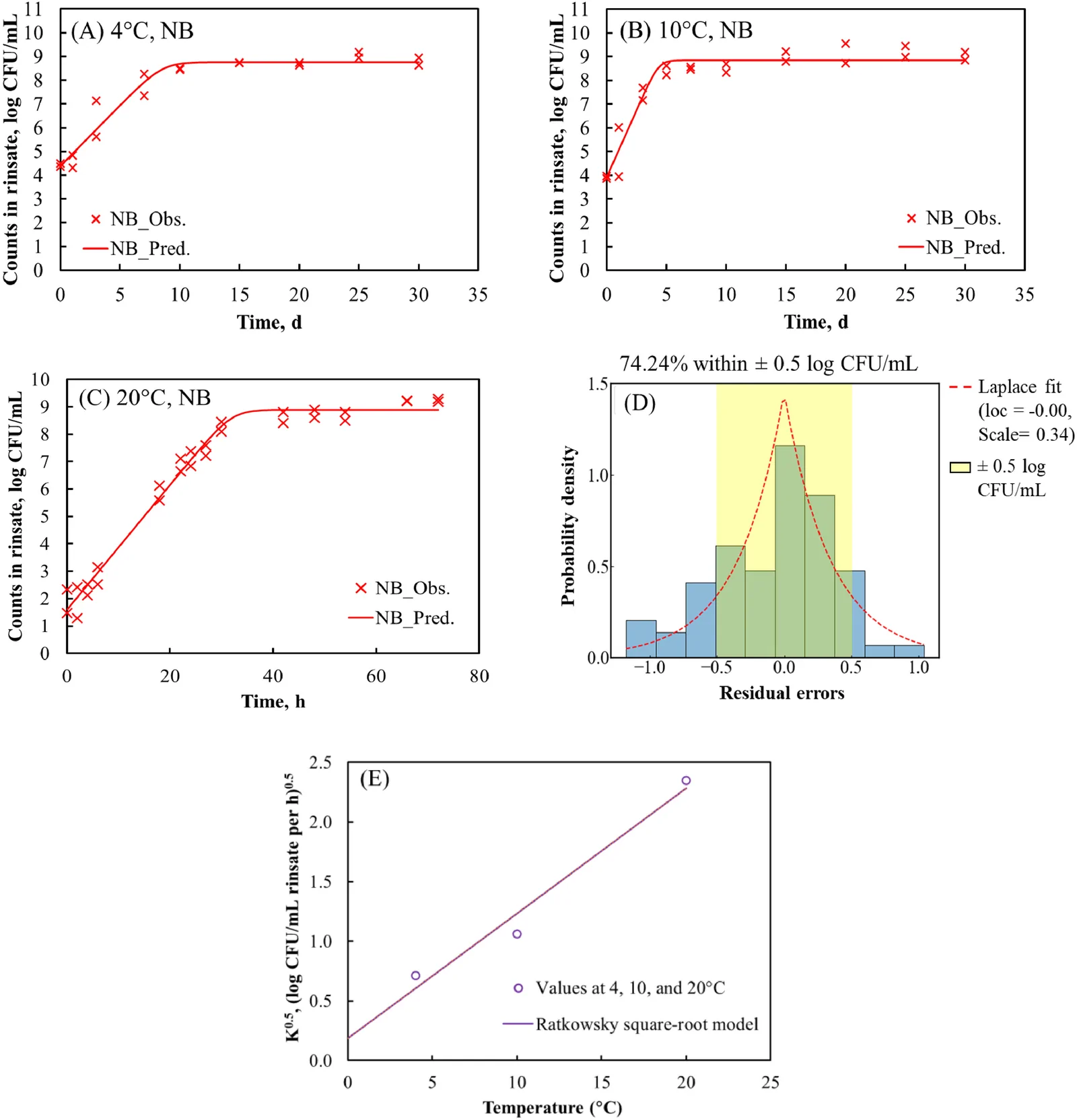
Growth and predicted curves of NB on chicken wings during storage at 4°C (A), 10°C (B), and 20°C (C). (D) Distribution of residual errors for the developed no lag model. (E) Specific growth rate prediction using the Ratkowsky square-root model. NB: Native bacteria; Symbols (×, ο) represent observed data, and solid red lines represent model predictions. Model-predicted values of Y(t), originally expressed as ln CFU/mL rinsate (Eq. (1)), were converted to log CFU/mL rinsate for plotting. K is the maximum growth rate in Eq. (2), which is calculated as µ_max_/2.203.

***Growth of NB on chicken wings and modeling.*** Fig. 1 shows the growth of NB on chicken wings during storage at 4, 10, and 20°C. The initial population of NB on chicken wings was approximately 4 log CFU/mL rinsate for the samples stored at 4 and 10°C (Fig. 1A and 1B) and approximately 2 log CFU/mL rinsate at 20°C (Fig. 1C). The starting-point bacterial concentrations in the samples stored at 4 and 10°C differed from those at 20°C, contributing to batch-to-batch variability in commercially sourced chicken wings. Under the experimental sampling conditions at 4, 10, and 20°C, lag phase was either not observed or practically negligible. This is due to the fact that NB are native to the wings and have already adapted to natural conditions. They did not need to adjust to the environment. Therefore, the no-lag phase model (Fang et al., 2013) was used to describe NB growth kinetics in this study. The estimated parameters of NB using the no-lag phase model are summarized in Table 1. At 4°C, NB populations increased gradually and reached the stationary phase at approximately 8–9 log CFU/mL rinsate after 10 d of storage from an initial load of ∼ 4 log CFU/mL. The growth of NB was well described by the no-lag phase model (Fig. 1A), yielding a small RMSE value of 0.4 log CFU/mL rinsate, indicating that the model was suitable for predicting NB growth at 4°C. The model estimated that the maximum growth rate (K, or $\mu_{max}/2.303$) of NB at 4°C was 0.51 log CFU/mL per d, and the maximum population was 8.8 log CFU/mL rinsate (Table 1), which means the bacterial population will increase by approximately 10 times every two days at this temperature.

**Table 1:** Estimated parameters for the no lag phase model and the secondary Ratkowsky square-root model. NB: Native bacteria. Y_0_ and Y_max_, originally expressed as ln CFU/mL rinsate (Eq. (1)), were converted to y_0_ and y_max_ in log CFU/mL rinsate. µ_max_ is converted to maximum growth rate K, or µ_max_/2.203, in log CFU/mL rinsate per d or per h (Eq. (1)).

NB	Parameters	Value	Std-error	t-value	*p*-value	L95CI	U95CI
No lag phase model							
4°C	y_0_, log CFU/mL rinsate	4.4	0.50	20.21	2.71E-12	9.06	11.19
	y_max_, log CFU/mL rinsate	8.7	0.32	62.42	1.53E-19	19.51	20.88
	K*, log CFU/mL rinsate per d	0.51	0.14	8.38	4.82E-07	0.88	1.48
	RMSE	0.4					
10°C	y_0_, log CFU/mL rinsate	3.9	0.69	13.11	2.56E-10	7.57	10.48
	y_max_, log CFU/mL rinsate	8.9	0.32	64.07	1.03E-21	19.72	21.06
	K*, log CFU/mL rinsate per d	1.13	0.37	6.97	2.26E-06	1.82	3.40
	RMSE	0.5					
20°C	y_0_, log CFU/mL rinsate	1.6	0.34	10.69	8.28E-11	2.96	4.38
	y_max_, log CFU/mL rinsate	8.9	0.27	74.98	5.98E-31	19.90	21.03
	K*, log CFU/mL rinsate per h	0.23	0.02	26.61	7.36E-20	0.48	0.57
	RMSE	0.5					
Ratkowsky square-root model for K*							
	a	0.10	0.02	5.62	0.11	−0.13	0.34
	T_0_,°C	−1.8					

* K = µ_max_/2.303.

At 10°C, NB growth followed a similar pattern, directly starting with exponential growth followed by a stationary phase (Fig. 1B). Compared to 4°C, NB grew faster at 10°C, with a higher maximum growth rate of 1.13 log CFU/mL per d (Table 1) and reached comparable stationary-phase levels of 8-9 log CFU/mL rinsate within a shorter storage period (approximately 6 d, starting from ∼ 4 log CFU/mL rinsate). At this temperature, the bacterial population would increase by more than 10 times per day. The no-lag growth model also provided a good fit to the observed data at 10°C (Fig. 1B), with an RMSE of 0.5 log CFU/mL rinsate. At 20°C, NB even grew much faster than at 4 and 10°C, with a maximum growth rate of 0.23 log CFU/mL per h (or 5.52 log CFU/mL per d) (Table 1), which means that the bacterial population will increase by approximately 10 times every 5 h. NB populations increased from approximately 2 log CFU/mL of rinsate to the stationary phase, reaching a maximum population of 8.9 log CFU/mL of rinsate within ∼37 h of storage. The good agreement between observed and predicted values at 20°C (Fig. 1C), with an RMSE of 0.5 log CFU/mL rinsate, further indicated the robustness of the no-lag phase model for predicting NB growth across diverse temperatures. The residual errors for the no-lag phase model followed a Laplace distribution, with a location parameter of −0.00 and a scale parameter of 0.34 (Fig. 1D). Approximately 74.2% of residual errors fell within ± 0.5 log CFU/mL rinsate, indicating that the majority of model deviations were within the reliability limits (± 0.5 log) of plate count–based enumeration (Corry et al., 2007) and supporting the suitability of the model for describing NB growth on chicken wings during storage at 4, 10, and 20°C.

There are many microorganisms native to chicken wings. Some are psychrotrophic. The observed growth of NB at both 4 and 10°C might be attributed to the presence and proliferation of psychrotrophic microorganisms in the chicken wings (Doulgeraki et al., 2012). Previous studies have shown that psychrotrophic bacteria, such as *Pseudomonas* spp. and lactic acid bacteria, grew well at 4 and 10°C (Balamatsia et al., 2006; Lee et al., 2017; Zhang et al., 2012), with total viable counts (**TVC**) increasing above ∼7-9 log CFU/g in chicken meat during storage at these temperatures (Balamatsia et al., 2006; Miks-Krajnik et al., 2016; Rukchon et al., 2014). The maximum growth rate of NB at 10°C was higher than that at 4°C, as a consequence of the temperature effect on bacterial growth rate (Bovill et al., 2001; Zhang et al., 2012). These results were in agreement with the findings reported by Miks-Krajnik et al., 2016, microbial maximum specific growth rates of 1.57 log CFU/g per d at 4°C and 2.26 log CFU/g per d at 10°C estimated using a modified Gompertz model on chicken meat during storage.

Fig. 1E exhibits the effect of temperature on the maximum growth rate (K) of NB described by the Ratkowsky square-root secondary model. The estimated model parameters are listed in Table 1. The estimated nominal minimum growth temperature (T_0_) of NB was −1.8°C, suggesting that NB growth would be minimal in near-freezing conditions. The value of T_0_ may vary among different studies due to differences in experimental conditions, food matrices, and the mathematical model used (Jia et al., 2020; Koutsoumanis, 2001; Lytou et al., 2016; Milkievicz et al., 2020; Zaher and Fujikawa, 2011). The positive slope parameter (a = 0.1) indicated a positive impact of temperature on NB’s maximum growth rate within the studied temperature range (≤ 20°C) in which NB grew faster as temperature increased (Table 1; Fig. 1E). Similar temperature-dependent growth patterns have been reported for indigenous microflora in poultry products (Milkievicz et al., 2020; Tarlak and Pérez-Rodríguez, 2022; Zaher and Fujikawa, 2011). These results highlight the importance of temperature control in managing spoilage microbial growth and product shelf life.

**Fig. 2:**
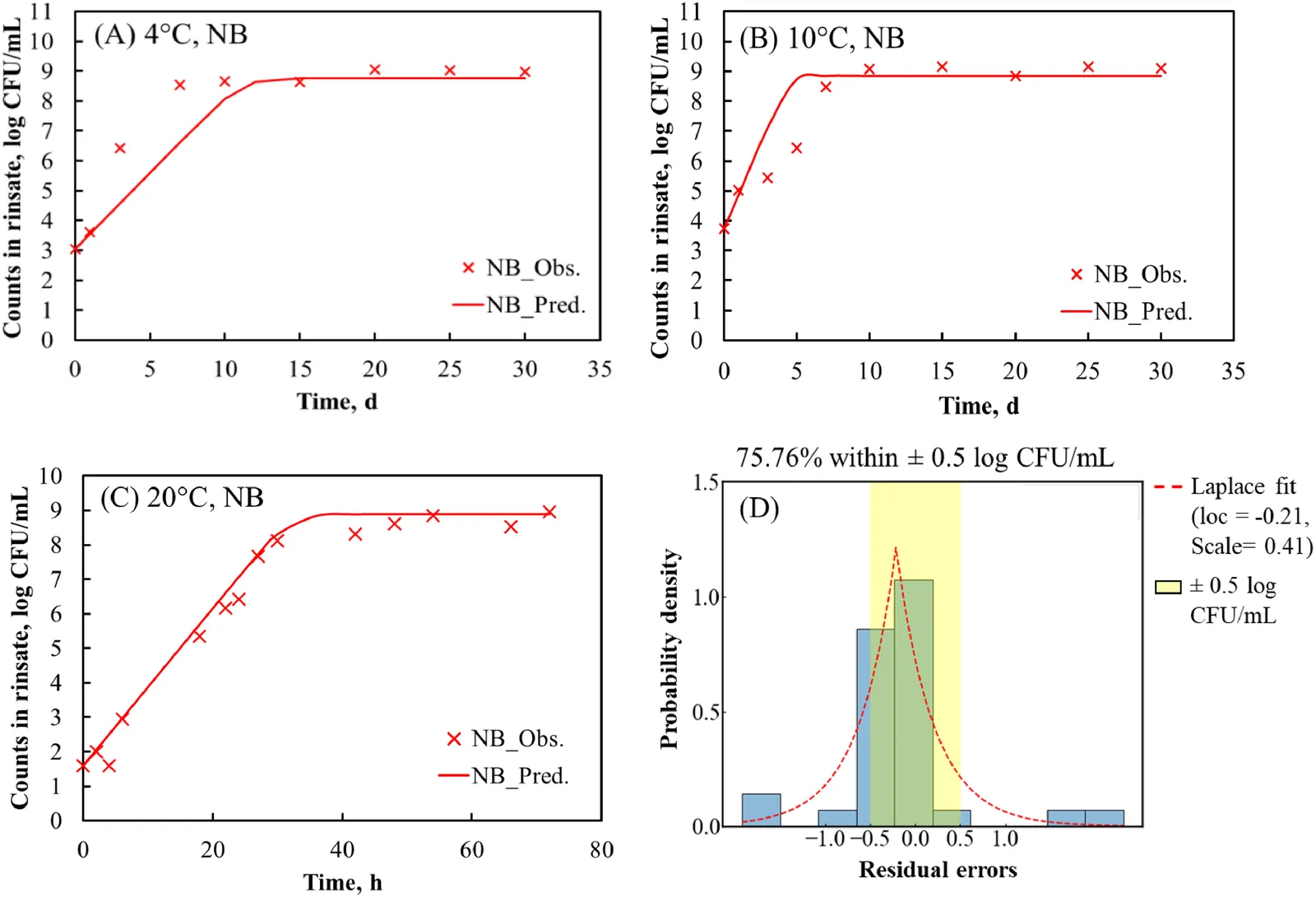
Validation of the no lag phase model for predicting NB growth on chicken wings during storage at 4°C (A), 10°C (B), and 20°C (C), and the distribution of residual errors for the model (D). Symbol (×) represents observed data, and the solid line represents model predictions. The dashed line indicates the fitted Laplace distribution, and the shaded area represents ± 0.5 log CFU/mL rinsate. NB: Native bacteria. Model-predicted values of Y(t), originally expressed as ln CFU/mL rinsate (Eq. (1)), were converted to log CFU/mL rinsate for plotting.

***Model validation for NB.*** The predictive performance of the developed no-lag phase model for predicting NB growth on chicken wings during storage at 4, 10, and 20°C was validated. The observed and predicted bacterial populations are presented in Fig. 2A-C, along with residual error distributions for the model (Fig. 2D). The no-lag phase model was able to capture the overall observed growth trend, as indicated by the alignment of predicted values throughout the storage period (Fig. 2A-C). Residual errors were symmetrically distributed around zero and followed a Laplace distribution (location = −0.21; scale = 0.41; Fig. 2D). Approximately 76% of predictions fell within ± 0.5 log CFU/mL rinsate of observed values (Fig. 2D), with RMSE values of 0.4-0.9 log CFU/mL rinsate. These results demonstrated that the no-lag phase model was acceptable to predict NB growth on chicken wings.

***NB growth simulation and chicken wing shelf-life prediction.*** NB populations in this study were quantified by plating on TSA followed by aerobic incubation, an accepted approach for determining the TVC (Augustynska-Prejsnar et al., 2024) or aerobic plate counts (**APC**) (Herron et al., 2022; Killinger et al., 2010; Lemonakis et al., 2017). A spoilage threshold of 7 log CFU/mL rinsate, based on TVC/APC, was adopted as the limit of acceptable microbiological quality for poultry meat (Dickens et al., 2004; Ikeme et al., 1982). Based on this threshold, the no-lag phase model and the Ratkowsky square-root model for NB were applied to simulate the microbial shelf life of chicken wings at different storage temperatures.

**Fig. 3:**
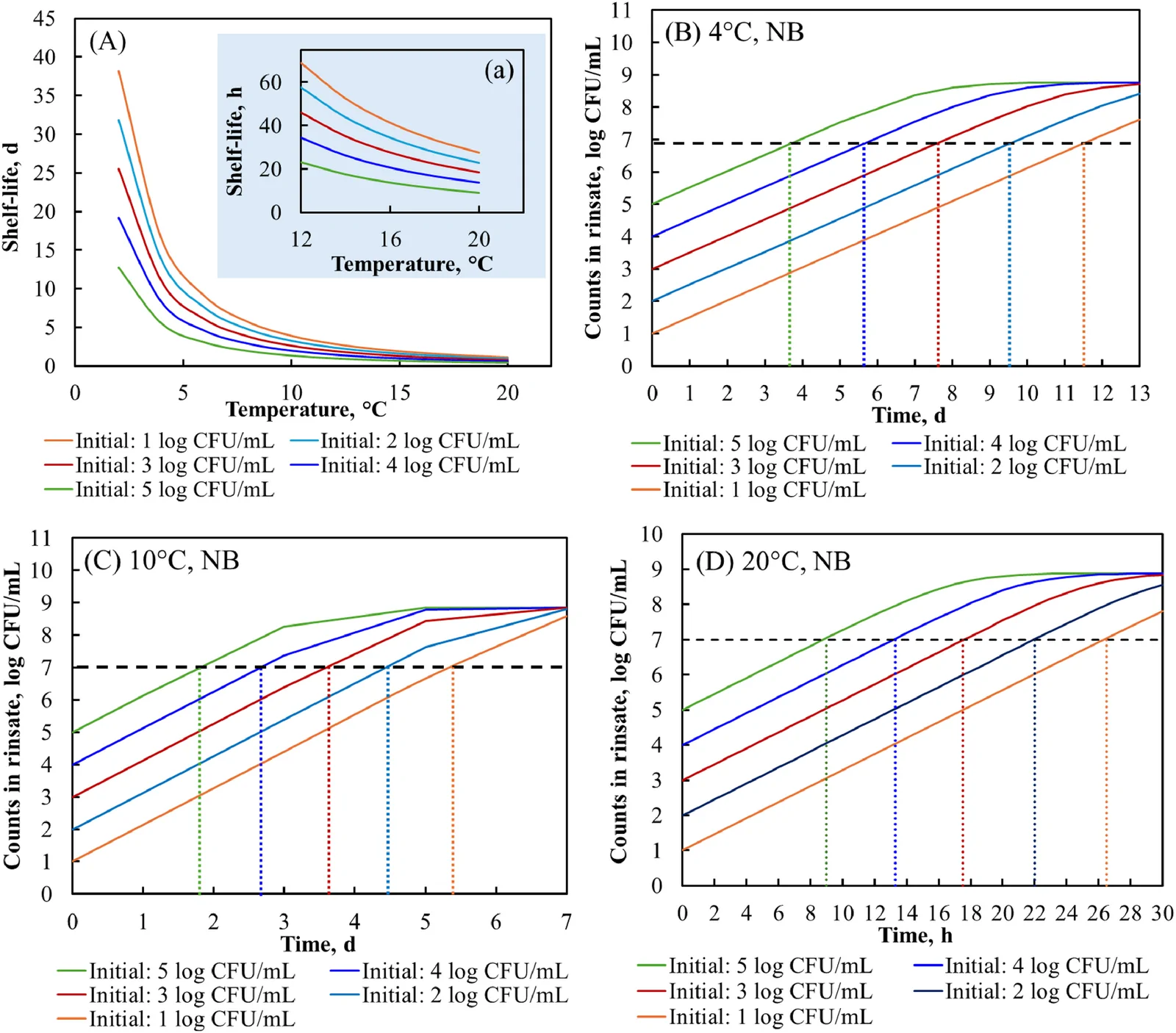
Simulation of NB growth on chicken wings during storage at 4, 10, and 20°C (A-C) using the developed no lag phase model, when initial levels of NB are set as 1, 2, 3, 4, and 5 log CFU/mL rinsate. (D) Effect of temperature on the shelf-life of chicken wings estimated by the no lag phase model and the Ratkowsky square-root model. Shelf life was predicted using 1, 2, 3, 4, and 5 log CFU/mL as initial NB levels, Y_max_ = 8.8 log CFU/mL rinsate (or 20.4 ln CFU/mL rinsate, average of maximum population of NB at 4, 10, and 20°C), and Y(t) = 7 log CFU/mL rinsate (or 16.1 ln CFU/mL rinsate, threshold for spoilage). The inset (a) shows the predicted shelf-life, expressed in hours, at temperatures ranging from 12 to 20°C. NB: Native bacteria. Model-predicted values of Y(t), originally expressed as ln CFU/mL rinsate (Eq. (1)), were converted to log CFU/mL rinsate for plotting.

**Fig. 4:**
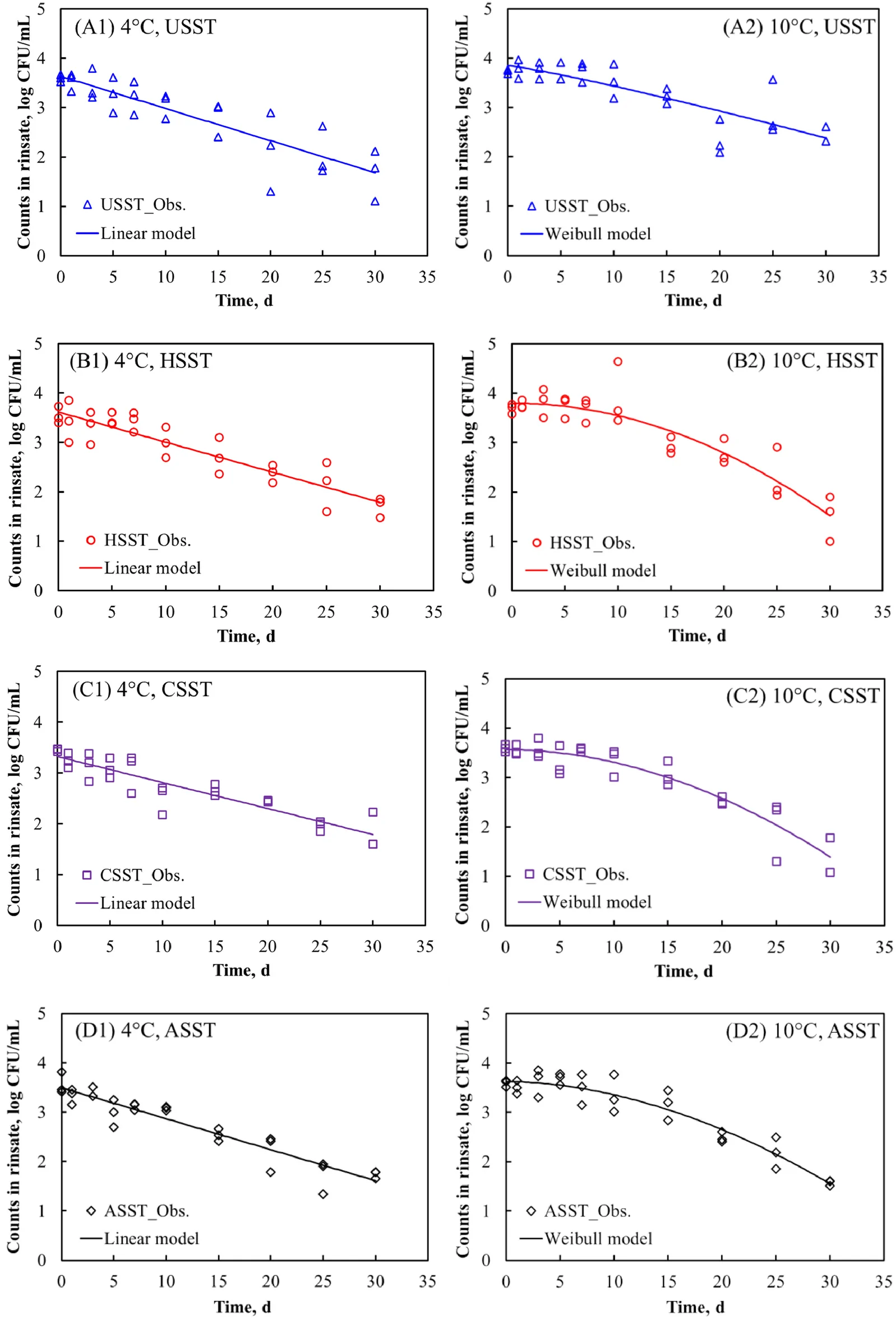
Survival curves and model fits for ST on chicken wings during storage at 4°C and 10°C. Panels A1-D1 show ST survival at 4°C described by the linear model, and panels A2-D2 show ST survival at 10°C described by the Weibull model. (A1-A2) USST, (B1-B2) HSST, (C1-C2) CSST, and (D1-D2) ASST. Symbols (Δ, o, □, ◊) represent observed data, and solid lines represent model fits. USST: Unstressed *Salmonella* Typhimurium; HSST: Heat-stressed *Salmonella* Typhimurium; CSST: Cold-stressed *Salmonella* Typhimurium; ASST: Acid-stressed *Salmonella* Typhimurium.

Model predictions demonstrated a temperature dependence of NB growth and shelf-life duration (Fig. 1A-C). At 4 and 10°C, when the initial NB concentration was around 4 log CFU/mL rinsate, NB populations reached this spoilage threshold after 5 d at 4°C (Fig. 1A) and 3 d at 10°C (Fig. 1B), suggesting the effect of temperature on the shelf life of chicken wings. At the abuse temperature of 20°C, starting from an initial NB concentration of around 2 log CFU/mL rinsate (Fig. 1C), the NB population reached the spoilage threshold of ∼ 7 log CFU/mL rinsate after approximately 23 h, demonstrating chicken wing spoilage within one day of exposure at 20°C. In addition, because the initial NB level can vary in chicken wings depending on processing and handling conditions, the time to reach the spoilage threshold may also differ. To account for these variabilities, the effect of temperature on the shelf life of chicken wings was simulated for shelf-life prediction across a range of initial NB levels of (1, 2, 3, 4, and 5 log CFU/mL rinsate, Fig. 3). The simulations revealed that shelf life is influenced by temperature and initial NB load (Fig. 3A). Lower initial NB levels and lower storage temperatures could extend the time needed to reach the spoilage threshold (7 log CFU/mL rinsate), resulting in longer shelf life (Fig. 3A). For example, chicken wings with an initial NB of 1 log CFU/mL rinsate were predicted to remain below the spoilage threshold for up to 12 d at 4°C (Fig. 3B) and 5.5 d at 10°C (Fig. 3C) whereas samples with an initial NB level of 3 log CFU/mL rinsate exceeded the limit after approximately 8 d at 4°C (Fig. 3B) and 4 d at 10°C (Fig. 3C). When chicken wings were exposed to a higher temperature of 20°C, the wings with an initial NB of 1 log CFU/mL rinsate were predicted to remain acceptable for up to 26 h (Fig. 3D), whereas those starting at 5 log CFU/mL rinsate exceeded the spoilage threshold after approximately 9 h (Fig. 3D). Overall, the integration of the no-lag phase model with the Ratkowsky square-root secondary model provides a data-driven framework for estimating the microbial shelf life of chicken wings with various initial microbial levels during storage at different temperatures (Fig. 3A); however, validation under commercial conditions is recommended before applying these predictions in practice.

### Survival of ST on chicken wings at 4 and 10°C

The growth of ST in chicken wings differed from NB. At 4 and 10°C, ST did not grow, and the population gradually declined. The decline was affected by stress. Fig. 4 presents the survival behavior of USST and stressed ST on chicken wings during storage at 4°C (Fig. 4A1-D1) and 10°C (Fig. 4A2-D2). The populations of USST and stressed ST on chicken wings exhibited decline trends over time during the 30-d storage at 4 and 10°C. By the end of the storage period, approximately 1–2 log CFU/mL rinsate of reduction was observed across USST and stressed ST. Reduction of *Salmonella* during storage at low temperatures (4-10°C) was reported in some studies (Silva et al., 2022). For instance, Argyri et al., 2018 reported around 1 log reduction of *Salmonella* on chicken meat after storage at 4°C for 14 d; Pradhan et al., 2012 observed a declining trend of *Salmonella* on chicken meat after storing 7 d at 4°C and 8°C. However, the reduction of ST populations observed at 4 and 10°C in the present study contrasted with some studies reporting survival or slow growth of *Salmonella* under similar temperature conditions (Al-Hijazeen et al., 2022; Noviyanti et al., 2024; Oscar, 2011, 2014; Zaher and Fujikawa, 2011). This difference is likely attributable to variations in experimental systems, microbial ecology, and strain-specific characteristics. It is highly unlikely that *Salmonella* could grow at 4°C, although it is possible that it may grow at 10°C, due to the fact that *Salmonella* is mesophilic. It is also normal that *Salmonella* growth may not occur at 10°C, which may be partially associated with the presence of NB. Chicken wings purchased from a local grocery store were kept at refrigerated temperature. NB on chicken wings may contain psychrotrophs that thrive at low temperatures*.* The unhindered growth of NB in Fig. 1 (Fig. 1A and 1B) suggests that NB might contribute to an unfavorable environment for ST growth, as they have already adapted to chicken wings.

At 4°C, both USST and stressed ST exhibited linear reduction trends. The linear model provided a good description of the observed decline patterns of USST, HSST, CSST, and ASST, with the estimated values matching experimental data (Fig. 4A1-D1). Table 2 lists the estimated model parameters. The relatively small RMSE values (< 0.3 log CFU/mL rinsate) for USST, HSST, CSST, and ASST (Table 2) demonstrated that the linear model was adequate to describe ST survival behavior at 4°C. The D values listed in Table 2 show the effect of treatment on bacterial survival at 4°C. The D values of USST, HSST, and ASST are very close to each (between 15.4 and 16.4 d), suggesting that the bacterial cells would decline by 90% about every two weeks. The D value of CSST, on the other hand, is 19.5 d, which is 3-4 d longer than the D values of USST, HSST, and ASST. It suggests that cold treatment may induce some kind of protection for ST, allowing the cells to withstand the cold storage better than unstressed, heat-stressed, and acid-stressed ST. Despite this difference in decline rate, the survival kinetics remained linear for all ST, suggesting that at 4°C, survival behavior might be governed primarily by low temperature, with stress influencing the rate of decline rather than changing the survival pattern.

**Table 2:** Estimated parameters of the linear and Weibull models for predicting ST survival on chicken wings during storage at 4°C and 10°C, respectively. USST: Unstressed *Salmonella* Typhimurium; HSST: Heat-stressed *Salmonella* Typhimurium; CSST: Cold-stressed *Salmonella* Typhimurium; ASST: Acid-stressed *Salmonella* Typhimurium.

Temp.	Treatment	Estimated parameters	Values	Std-error	t-value	*p*-value	L95 CI	U95 CI
4°C	USST	y_0_, log CFU/mL rinsate	3.6	0.10	35.95	5.60E-25	3.43	3.84
		D, d	15.4	1.57	9.83	1.41E-10	12.18	18.59
		RMSE	0.3					
	HSST	y_0_, log CFU/mL rinsate	3.6	0.08	45.60	8.04E-28	3.46	3.78
		D, d	16.4	1.40	11.73	2.54E-12	13.55	19.28
		RMSE	0.3					
	CSST	y_0_, log CFU/mL rinsate	3.3	0.07	51.17	3.31E-29	3.19	3.46
		D, d	19.5	1.63	12.03	1.45E-12	16.23	22.87
		RMSE	0.2					
	ASST	y_0_, log CFU/mL rinsate	3.5	0.07	53.48	6.18E-29	3.36	3.63
		D, d	15.9	1.07	14.93	1.44E-14	13.72	18.10
		RMSE	0.3					
10°C	USST	y_0_, log CFU/mL rinsate	3.8	0.14	28.28	1.69E-20	3.56	4.12
		D, d	21.5	3.42	6.29	1.39E-06	14.49	28.59
		α	1.1	0.39	2.98	6.40E-03	0.35	1.95
		RMSE	0.3					
	HSST	y_0_, log CFU/mL rinsate	3.8	0.10	37.52	7.82E-25	3.59	4.01
		D, d	19.9	1.75	11.36	8.61E-12	1.63	23.49
		α	2.0	0.41	4.87	4.32E-05	1.17	2.86
		RMSE	0.3					
	CSST	y_0_, log CFU/mL rinsate	3.6	0.08	44.54	4.44E-26	3.40	3.73
		D, d	20.2	1.42	14.17	9.64E-14	17.23	23.07
		α	1.9	0.03	5.70	5.42E-06	1.25	2.65
		RMSE	0.2					
	ASST	y_0_, log CFU/mL rinsate	3.6	0.07	52.39	1.08E-28	3.48	3.77
		D, d	20.3	1.28	15.91	3.08E-15	17.66	22.89
		α	1.9	0.28	6.71	3.34E-07	1.29	2.43
		RMSE	0.2					

In contrast to the linear survival curves observed at 4°C, the survival curves at 10°C were all nonlinear for stressed ST cells, except for USST, suggesting the refrigerated storage temperature may not affect the kinetics of bacterial survival for USST. At 10°C, the population of USST would decline by 90% about every 21 d, following the linear kinetics (shape parameter (α) ≈ 1; Table 2). However, the survival curves of stressed cells at 10°C were downwardly concave and matched well to the Weibull model (Fig. 4A2-D2). The RMSEs of the fitted curves are 0.2-0.3 log CFU/mL rinsate for the stressed cells (Table 2), indicating a good model fit. All estimated parameters showed statistically significant (*p* < 0.05) (Table 2). Since the survival curves of stressed ST are downwardly concaved, the shape parameter (α) all > 1, estimated as 2.0, 1.9, and 1.9 for HSST, CSST, and ASST. The shape factor affects the shape of the Weibull model. A larger value (> 1) suggested a higher degree of deviation from the linear model. Given the fact that the shape parameters are very close to each other (∼2), the survival curves of HSST, CSST, ASST, and HSST bend downward in a similar manner away from the linear kinetics.

The curves of stressed ST all showed a slow decline at the early stage of storage, followed by a gradually accelerated decrease in the population as storage progressed. Since the shape parameters of the survival curves are almost the same, it is possible to compare the effect of time on bacterial survival directly. The slow initial decline is manifested by the large first decimal deduction time in each treatment, which is 19.9 d for HSST, 20.2 d for CSST, and 20.3 d for ASST. Therefore, storage at 10°C for about 20 d would lead to 90% decrease in the stressed ST. For USST, the population of USST would decrease by 1.5 log CFU/mL rinsate from an initial level of 3.8 log CFU/mL after 30 days of storage at 10°C. However, stressed ST, the population would decrease by 2 log CFU/mL rinsate. This indicated that although stressed ST cells initially persisted better during the initial storage period, their populations declined faster at later stages than USST. The increased shape parameters (α > 1) for stressed ST compared to USST (α ≈ 1) may indicate some protective mechanisms activated by the stress. Cold stress protective mechanisms might be associated with cell membrane modification, altered gene expression and metabolism, and cold-shock protein synthesis (Ricke et al., 2018; Shah et al., 2013). Acid stress might trigger the acid‐adaptive response, which activates the defense mechanism (e.g., acid shock protein synthesis, gene expression regulation, and changes in metabolic activity and membrane fluidity) to improve survival ability (Alvarez-Ordóñez et al., 2012; Foster, 2001; Osaili et al., 2017; Suehr et al., 2020; Xu et al., 2008). Prior exposure to heat stress likely activates stress response systems that alter gene expression, cellular metabolism, heat shock protein activity, and other protective pathways that improve cellular maintenance and repair functions, thereby increasing the likelihood of their survival (Dawoud et al., 2017; Spector and Kenyon, 2012).

These results at 4 and 10°C demonstrated that the impact of stress on ST survival kinetics is temperature- and stressor-dependent, which aligned with the reported findings (Osaili et al., 2017). Stressed ST exhibited linear survival kinetics at 4°C, while displaying non-linear survival kinetics at 10°C. Additionally, at 4 and 10°C, the time to achieve 90% bacterial reduction was not uniform across stresses and temperatures. These findings highlighted the need to account for both stress and storage temperature factors when developing predictive models and conducting risk assessments for *Salmonella* in poultry products.

### Growth of USST and stressed ST at 20°C

**Fig. 5:**
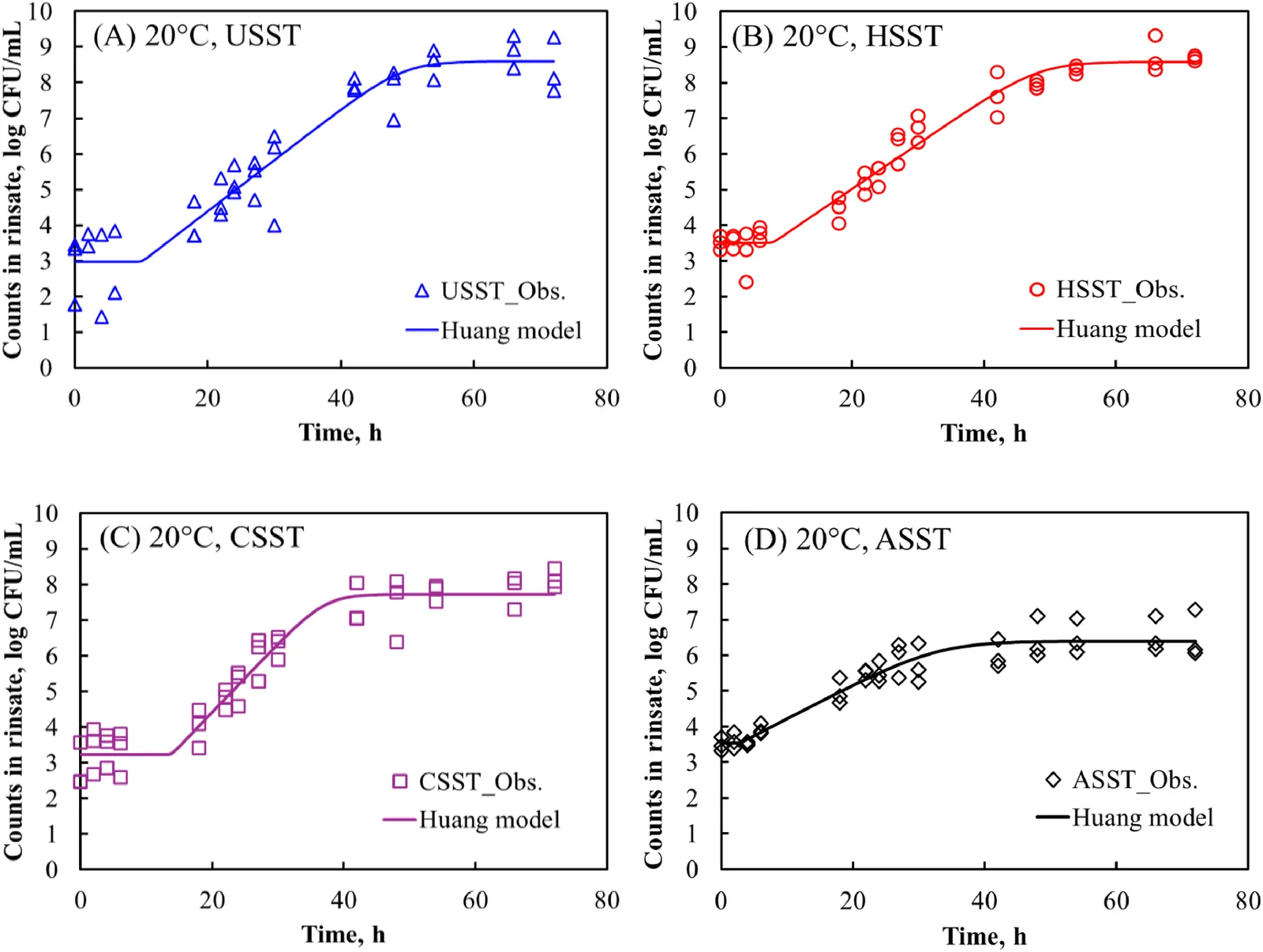
Experimental data of USST and stressed ST on chicken wings during storage at 20°C and corresponding fits of the Huang model (A-D). USST: Unstressed *Salmonella* Typhimurium; HSST: Heat-stressed *Salmonella* Typhimurium; CSST: Cold-stressed *Salmonella* Typhimurium; ASST: Acid-stressed *Salmonella* Typhimurium. Symbols (Δ, o, □, ◊) represent observed data, and solid lines represent model fits. Model output values of Y(t), originally expressed as ln CFU/mL rinsate (Eq. (5)), were converted to log CFU/mL rinsate for plotting.

Fig. 5A-D displays the growth behaviors of USST and stressed ST on chicken wings during storage at an abuse temperature of 20°C and corresponding fits of the Huang model. The estimated growth kinetic parameters using the Huang model are summarized in Table 3. The initial levels of inoculated USST and stressed ST were around 3 log CFU/mL rinsate (Fig. 5A-D). After storing 72 h at 20°C, USST, and stressed ST grew well on chicken wings, displaying distinct lag, exponential, and stationary phases (Fig. 5A-D). As shown in Fig. 5A-D, the Huang model adequately described the experimental data, as indicated by small RMSE values of 0.5, 0.4, 0.6, and 0.3 log CFU/mL rinsate for USST, HSST, CSST, and ASST, respectively (Table 3).

**Table 3:** Estimated kinetic parameters for USST and stressed ST in chicken wings during storage at 20°C using the Huang model. USST: Unstressed *Salmonella* Typhimurium; HSST: Heat-stressed *Salmonella* Typhimurium; CSST: Cold-stressed *Salmonella* Typhimurium; ASST: Acid-stressed *Salmonella* Typhimurium. Y_0_ and Y_max_ (Eq. (4)), originally expressed as ln CFU/mL rinsate (Eq. (1)), were converted to y_0_ and y_max_ in log CFU/mL rinsate; λ: lag phase duration; µ_max_: Specific growth rate. Model-predicted values of Y_0_ and Y_max_, originally expressed as ln CFU/mL rinsate (Eq. (4)), were converted to log CFU/mL rinsate. µ_max_ is converted to maximum growth rate K, or µ_max_/2.203, in log CFU/mL rinsate per h (Eq. (5)).

	Parameters	Values	Std-error	t-value	*p*-value	L95CI	U95CI
USST	y_0_, log CFU/mL rinsate	3.0	0.55	12.51	1.80E-14	5.75	7.98
	λ, h	10.1	3.16	3.19	3.00E-03	3.66	16.47
	y_max_, log CFU/mL rinsate	8.6	0.59	33.55	3.34E-28	18.59	20.98
	K*, log CFU/mL rinsate per h	0.14	0.04	7.40	1.18E-08	0.24	0.42
	RMSE	0.5					
HSST	y_0_, log CFU/mL rinsate	3.5	0.27	29.90	5.17E-28	7.50	8.59
	λ, h	7.9	2.18	3.64	8.20E-04	3.51	12.35
	y_max_, log CFU/mL rinsate	8.6	0.33	59.56	3.91E-39	19.08	20.43
	K*, log CFU/mL rinsate per h	0.13	0.03	10.61	6.42E-13	0.24	0.35
	RMSE	0.4					
CSST	y_0_, log CFU/mL rinsate	3.2	0.34	21.75	4.76E-23	6.77	8.16
	λ, h	13.8	2.02	6.84	4.03E-08	9.74	17.92
	y_max_, log CFU/mL rinsate	7.7	0.32	56.46	2.91E-38	17.18	18.46
	K*, log CFU/mL rinsate per h	0.19	0.08	5.94	6.80E-07	0.29	0.60
	RMSE	0.6					
ASST	y_0_, log CFU/mL rinsate	3.5	0.37	22.35	1.84E-23	7.43	8.91
	λ, h	2.8	2.45	1.17	2.51E-01	−2.10	7.80
	y_max_, log CFU/mL rinsate	6.4	0.24	62.18	7.74E-40	14.27	15.24
	K*, log CFU/mL rinsate per h	0.10	0.02	9.34	2.19E-11	0.17	0.27
	RMSE	0.3					

* K = µ_max_/2.303.

As shown in Fig. 5A-D, environmental stresses (heat, cold, and acid) exhibited influences on the growth kinetics of ST on chicken wings during storage at 20°C, which was a temperature representing severe temperature abuse and would allow observable growth of ST to occur. The lag phase of USST was 10.1 h, with a maximum growth rate of 0.14 log CFU/mL rinsate per h, and a maximum population of 8.6 log CFU/mL rinsate (Fig. 5A; Table 3). Oscar reported that experimental and least-squares regression model-predicted unstressed *Salmonella* Typhimurium DT104 on chicken skin stored at 20°C remained in the lag phase for 8 h or longer (Oscar, 2009a). Some studies have also reported varying growth kinetic parameter values for *Salmonella* on chicken meat at 20°C (Milkievicz et al., 2020), such as maximum growth rates of approximately 0.5 log CFU/g per h of *Salmonella* cocktail predicted using a modified Gompertz model in raw chicken tenderloins (Juneja et al., 2007), 0.6 log CFU/g per h of *Salmonella* Typhimurium SL1344 estimated using a Baranyi model in ground chicken meat (Zhou et al., 2014), 1.6 log CFU/g per h of rifampicin-resistant *Salmonella* cocktail predicted using a Huang-Jameson effect model in ground chicken (Jia et al., 2020), 0.2 log CFU/cm^2^ per h of antibiotic-resistant *Salmonella* Kentucky estimated using a Baranyi model in raw chicken tenderloins (Dominguez and Schaffner, 2008), and 0.1 log CFU/g per h of *Salmonella* Typhimurium DT104 predicted using a logistic with delay model in ground chicken breast meat (Oscar, 2006), which differ from the value obtained in this study. Such variability in growth parameters may be attributed to several factors, including: 1) differences in *Salmonella* strains (e.g., different strains, antibiotic-resistant vs. non-resistant strains, or single-strain vs. multi-strain cocktail) (Dominguez and Schaffner, 2008; Lianou and Koutsoumanis, 2011, 2013); 2) the type of chicken matrix used (e.g., ground chicken, tenderloin, and chicken wings) as physical structure, nutrient availability, and surface properties differ among them (Dominguez and Schaffner, 2008; Juneja et al., 2007; Noviyanti et al., 2024); 3) initial pathogen inoculation levels (Oscar, 2006, 2007), and 4) the mathematical modeling approach employed for kinetic parameter estimation (Dominguez and Schaffner, 2008; Jia et al., 2020; Juneja et al., 2007).

The growth kinetics of HSST on chicken wings differed slightly from those of USST when exposed to 20°C (Fig. 5A and 5B; Table 3). The lag phase of HSST was 7.9 h, approximately 2 h shorter than that of USST (10.1 h), while their maximum growth rates were comparable (0.13 and 0.14 log CFU/mL rinsate per h, respectively) (Table 3). The maximum population of HSST reached 8.6 log CFU/mL rinsate, which was the same as that of USST (8.6 log CFU/mL rinsate) (Table 3), indicating that heat stress had no effect on overall biomass. The reduced lag phase in HSST might result from the activation of multiple physiological responses induced by heat stress, including upregulation of certain gene transcriptions, synthesis of various heat shock proteins (Roncarati and Scarlato, 2017), and regulation of signaling pathways (Gottesman, 2019; Hamill et al., 2020). These responses likely aid in bacterial multiplication and promote early cell division. Some studies have reported that heat stress could prolong the lag phase because injured cells generated from heat stress need repair time to recover from injury before resuming multiplication (Mackey and Derrick, 1982; Wesche et al., 2009). It has also reported no effect of stress on the lag phase (Pin et al., 2012). The discrepancy between our findings and these reports may be attributed to differences in pathogen strains (Aguirre et al., 2014; Hamill et al., 2020), heat stress parameters (e.g., temperature and duration) (Aguirre et al., 2014; Pin et al., 2012), and the type of recovery media used (non-selective media vs. selective media) (Stephens et al., 1997; Wesche et al., 2009). Though the lag phase can be affected by heat stress, it does not necessarily affect the exponential growth rate, consistent with previous observations that lag duration and growth rate are not always correlated (Hamill et al., 2020; Pin et al., 2012; Wesche et al., 2009).

Cold stress also exhibited impacts on *Salmonella* growth kinetics compared to USST during storage at 20°C (Fig. 5A and 5C; Table 3). CSST had an extended lag phase of 13.8 h, approximately 4 h longer than USST (10.1 h), and demonstrated a slightly increased maximum growth rate (0.19 log CFU/mL rinsate per h) compared to USST (0.15 log CFU/mL rinsate per h) (Table 3). The maximum population of CSST (7.7 log CFU/mL rinsate) was around 1 log lower than that of USST (8.6 log CFU/mL rinsate) (Table 3). The prolonged lag phase in CSST was consistent with the study of Di Pasqua et al. (Di Pasqua et al., 2013). The prolongation of the lag phase and changes in maximum growth rate and maximum population might be attributed to changes in gene expression (e.g., DnaK) (Di Pasqua et al., 2013) and major cold shock protein production from cold stress (Berry and Foegeding, 1997; Horton et al., 2000), influencing the repair of molecular damage, metabolic processes, and the synthesis of cellular components necessary for growth (Ricke et al., 2018; Rolfe et al., 2012; Shah et al., 2013). Like heat stress, cold stress revealed a disconnect between the lag phase and the maximum growth rate.

It is worth noting that the lag phase of ASST on chicken wings was substantially shortened to 2.8 h, approximately 4-fold shorter than that of USST (10.1 h) (Fig. 5A and 5D; Table 3), suggesting that acid-stressed cells initialized growth much more rapidly on chicken wings following exposure to 20°C. ASST displayed a slightly reduced maximum growth rate (0.10 log CFU/mL rinsate per h) in comparison with USST (0.15 log CFU/mL rinsate per h). The maximum population of ASST (6.4 log CFU/mL rinsate) was approximately 2 log CFU/mL rinsate less than USST (8.6 log CFU/mL rinsate) (Table 3). These differences in growth kinetics between ASST and USST might be due to the induction of acid tolerance response (Alvarez-Ordóñez et al., 2010), alterations in gene expression, and synthesis of acid shock proteins in response to acid stress (Badie et al., 2021; Hamill et al., 2020).

These findings in USST and stressed ST demonstrated that environmental stresses could alter the growth kinetics of ST on chicken wings when exposed to 20°C, and these changes have direct implications for microbial risk modeling. Stress-induced changes in lag phase, growth rate, and maximum population affect early- and late-stage growth predictions, demonstrating that the physiological state of the cells should be incorporated for predictive model development. Models relying solely on USST may underestimate early growth (as in ASST) or mischaracterize the initiation of exponential growth (as in CSST). Incorporating environmental stresses in the predictive model development would improve model accuracy and enhance risk prediction in poultry products.

In this study, only a single strain of a single *Salmonella* serovar was evaluated. However, variations among *Salmonella* serovars, strains, and sources of isolation may influence bacterial survival and growth responses to stresses. Therefore, the findings and the estimated survival/growth kinetic parameters developed in this study should be interpreted primarily for the specific ST strain under the evaluated conditions. Future studies should investigate stress responses using multiple *Salmonella* strains representing diverse serovars and isolation sources to better understand the impact of stress on *Salmonella* behaviors. In addition, the ST strain used in this study was induced to NA resistance. Though the NA-resistant ST was induced and used to distinguish inoculated cells from native *Salmonella,* and all unstress and stress treatments were conducted using the same NA-resistant strain, the potential impact of NA-resistant inductions on the ST strain was not assessed in this study. Comparing the NA-resistant strain and the parental ST strain should be conducted in future work. Furthermore, chicken wings used in this study contained NB; however, the composition of NB was not characterized, and the relationship between NB and ST was not evaluated; therefore, further studies are needed to identify NB compositions and determine the interaction between NB and ST during storage. The unit of bacterial enumeration used in this study was log CFU/mL rinsate, while other units such as log CFU/g or log CFU/cm^2^ were also reported in many poultry microbiological studies (Mehmetoglu, 2011; Yang et al., 2001; Oscar, 2011). The use of different units is primarily dependent on the food matrix and sampling methodology. This unit has also been reported in previous poultry microbiological studies using similar bacterial quantification approaches (Bourassa et al., 2021; Incili et al., 2020; Kataria et al., 2020; Thanissery and Smith, 2014). Although differences in enumeration units may limit direct quantitative comparisons among studies, the results within this study remain comparable because the same inoculation, sampling, and enumeration procedures were consistently applied. Therefore, when comparing results with other studies, differences in sampling methods and reporting units should be considered. Additionally, different models were applied to describe ST behavior at different storage temperatures because the observed kinetic patterns varied with temperature. At 4°C, ST populations showed a linear survival pattern and were described using the linear model. At 10°C, stressed ST exhibited nonlinear survival patterns, and the Weibull model provided a better description of the observed behavior. At 20°C, ST demonstrated growth behavior, which was characterized using the Huang model. These models were used as descriptive kinetic models to characterize ST behavior and estimate kinetic parameters under the evaluated conditions rather than to predict ST responses beyond the experimental dataset. Future studies may consider applying machine learning approaches to integrate multiple influencing factors, including temperature, stress history, and microbial responses, to develop more robust predictive models for ST behavior in poultry products.

## Conclusions

This study demonstrated that the growth and survival of ST on chicken wings were impacted by stresses (heat, cold, and acid) and temperatures. Under refrigerated conditions (4 and 10°C), USST and stressed ST populations declined during 30-d storage, whereas growth behavior was observed at 20°C. Stress effects on ST behavior were demonstrated to be temperature- and stressor-dependent. At 4°C, both USST and stressed ST exhibited linear reduction kinetics, with the D value of CSST being 3-4 d longer than those of USST, HSST, and ASST. At 10°C, USST maintained a linear pattern, whereas stressed ST displayed nonlinear kinetics with shape parameters higher than 1, suggesting the protective mechanisms in stressed ST. At 20°C, ST growth kinetic parameters (lag phase, maximum growth rate, and maximum population) were affected by stress, particularly for ASST. Additionally, no-lag phase and Ratkowsky square-root models were developed to describe NB growth and predict chicken wing shelf life. These results indicated the importance of incorporating the factors of stress, temperature, and native microbiota when developing future predictive models to improve the reliability and accuracy of pathogen risk assessment. Future research should investigate the effects of stress across multiple *Salmonella* strains, under dynamic temperature conditions, assess potential impacts on virulence and pathogenicity, and develop integrated models capturing these interacting factors to enhance predictive accuracy and support effective food safety interventions in poultry products.

## Disclosures

The authors declare that they have no known competing financial interests or personal relationships that could have appeared to influence the work reported in this paper.
